# Neurosarcoidosis With Multi-Organ Involvement: A Case Report and Literature Review

**DOI:** 10.7759/cureus.43254

**Published:** 2023-08-10

**Authors:** Nicolas Naccarella, Samia Ikhlef, Jacques Rommens

**Affiliations:** 1 Department of Radiology/Interventional Radiology, Hôpital Universitaire de Bruxelles (H.U.B), Brussels, BEL; 2 Department of Radiology, Hôpital Universitaire de Bruxelles (H.U.B), Brussels, BEL; 3 Department of Radiology/Interventional Radiology, Hôpital Delta, Chirec, Brussels, BEL

**Keywords:** neuro mri, central nervous system disease, nodular leptomeningeal enhancement, walking disorder, headaches, neurosarcoidosis

## Abstract

Sarcoidosis is a multisystemic disease that, in rare cases, can involve the central nervous system (CNS). We present a case of sarcoidosis with intracranial and multi-organ involvement. The patient presented with a one-month history of headaches. Imaging revealed leptomeningeal nodular enhancement (LNE), and a PET/CT scan of the chest and abdomen showed bilateral hilar, retroperitoneal, and inguinal lymphadenopathy. The diagnosis of sarcoidosis was confirmed by an ultrasound-guided inguinal lymph node biopsy. The patient was started on a combination of corticosteroids and immunosuppressive drugs, with a gradual improvement in symptoms and radiological findings over several months.

## Introduction

Sarcoidosis is a multisystemic inflammatory disorder characterized by the formation of granulomas in various organs and tissues, which can cause inflammation and damage [1].

Neurosarcoidosis is a rare form of sarcoidosis in which the granulomas, or clusters of inflammatory cells, affect the central nervous system (CNS), including the brain, spinal cord, and peripheral nerves [2,3]. Neurosarcoidosis may occur in isolation or as part of multisystemic sarcoidosis, a disorder involving multiple organ systems [3]. Neurosarcoidosis with multisystemic involvement is estimated to occur in 5%-15% of people [4].

The presentation of neurosarcoidosis varies widely and depends on the location and extent of the CNS involvement. The most common symptoms of neurosarcoidosis include headache, facial weakness, vision loss, hearing loss, seizures, and cognitive impairment [5]. The onset of symptoms is usually gradual, but in some cases, neurosarcoidosis can present as an acute neurological emergency, with seizures or stroke-like symptoms [6].

We report a patient presenting with chronic progressive worsening headaches, with anisocoria and dysmetria, who was found to have bilateral hilar, inguinal, and retroperitoneal lymphadenopathy and interstitial pulmonary nodules and testicular nodules on imaging. To our knowledge, the present case with the co-occurrence of these distinct manifestations is the first reported.

## Case presentation

A 27-year-old patient was admitted to the emergency department for mild continuous headaches for one month, worsening in the last 48 hours. His recent past history included gait disorder and dysarthria for three months. He had a similar episode one year ago but with spontaneous remission. The patient has no other medical, surgical, or family history. He was not receiving any medication.

During the general clinical examination, the patient was alert, oriented, and responsive. A cardiovascular examination revealed a regular rhythm without murmurs. Lungs were clear to auscultation bilaterally, and the abdomen was soft, non-tender, and non-distended, with no palpable masses or hepatosplenomegaly.

On neurological examination, cranial nerve examination was unremarkable, except for poorly reactive anisocoria to light (Adie's pupil). The motor assessment showed normal muscle tone, strength, and bulk without any focal deficits. Sensory examination revealed intact sensation to light touch, pain, temperature, and vibration. Deep tendon reflexes were symmetric and within normal limits. However, cerebellar tests revealed dysmetria on the finger-to-nose test and an ataxic gait characterized by an enlargement of the sustentation polygon in a standing position. He had difficulties walking in a straight line. His speech was slurred, "scanning," and irregular, showing cerebellar dysarthria. Plantar reflexes were flexor bilaterally.

The initial blood work, which included a complete blood count and comprehensive metabolic panel, showed results within the normal range. The C-reactive protein level was also normal (1.3 mg/dL). The results of serological tests for HIV, cytomegalovirus, toxoplasmosis, and Bartonellosis were all negative. Tuberculin skin testing was also negative. Antineutrophil cytoplasmic antibody (ANCA) serology was negative, with a titer of 20 (negative if <40). Anti-myeloperoxidase (MPO) antibodies and anti-proteinase 3 (PR3) antibodies were negative at 10 U/mL (positive if >25 U/mL and strongly positive if >80 U/mL). There were no elevated levels of serum IgG4 (<1.35 g/L). Serum angiotensin-converting enzyme (ACE) was also normal.

The patient had a lumbar puncture procedure. Cerebrospinal fluid (CSF) analysis revealed lymphocyte-predominant pleocytosis with no elevated ACE level of 100 cells/μL, 86% lymphocyte, glucose of 64 mg/dL, protein of 101 mg/dL, and CSF ACE of 1.4 U/L. CSF cytology test results were negative for meningeal carcinomatosis and showed no malignant cells. The presence of oligoclonal bands was not detected. CSF culture was negative for *Mycobacterium tuberculosis* and fungal infections.

**Figure 1:**
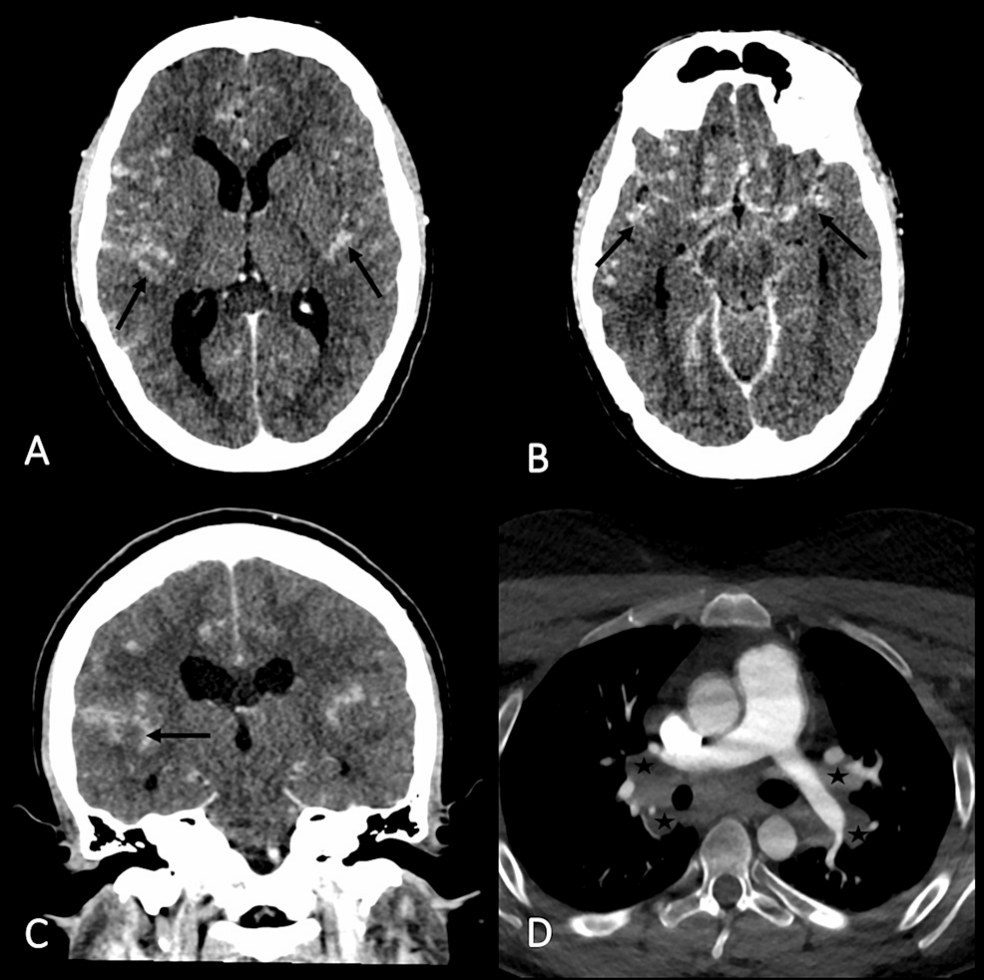
Brain CT scan with nodular leptomeningeal enhancement and lymphadenopathies in the upper part of the mediastinum Initial venous CT showing multiple hyperdensities in cerebral hemispheres predominantly in the temporal lobes corresponding to nodular leptomeningeal enhancement (black arrows) displayed with axial (A,B) and coronal (C) planes. It refers to the presence of nodules or areas of increased contrast enhancement in the leptomeninges. The arterial phase included the upper part of the mediastinum that demonstrated hilar lymphadenopathies (black stars) (D). CT: computed tomography

A three-phase CT of the brain (no contrast, arterial and venous phases) was obtained to exclude stroke or venous sinus thrombosis at the emergency department. It demonstrated in the venous phase a diffuse leptomeningeal nodular enhancement (LNE) (Figure 1A-1C). No anomaly was detected in the brain in the other phases. The arterial phase showed hilar lymphadenopathies (Figure 1D).

**Figure 2:**
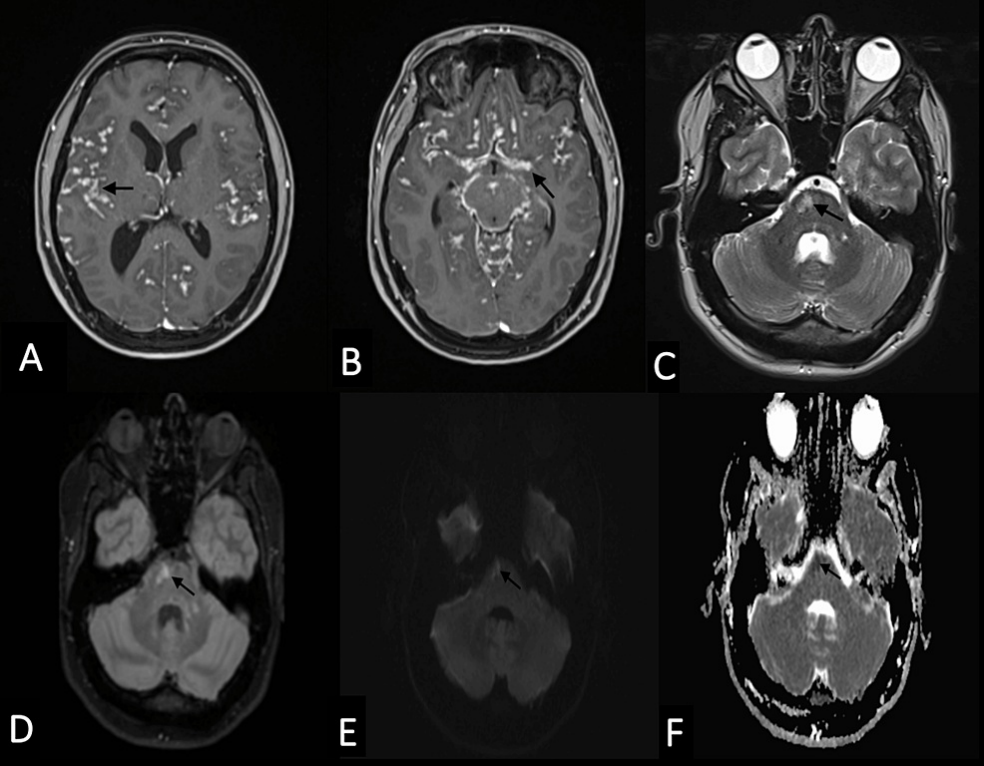
Brain MRI MRI showing the leptomeningeal nodular enhancement (black arrows) displayed on post-contrast axial T1WI (A,B). Recent pontic and brain stem ischemic lesions are demonstrated (black arrows), characterized by a hypersignal on sequences axial T2 (C) and axial FLAIR (D) with diffusion restriction (E,F). MRI: magnetic resonance imaging, T1WI: T1-weighted image, FLAIR: fluid-attenuated inversion recovery

MRI confirmed the leptomeningeal nodular enhancement (Figure 2A, 2B) and ischemic lesions localized in the right side of the pons (Figure 2C-2F).

**Figure 3:**
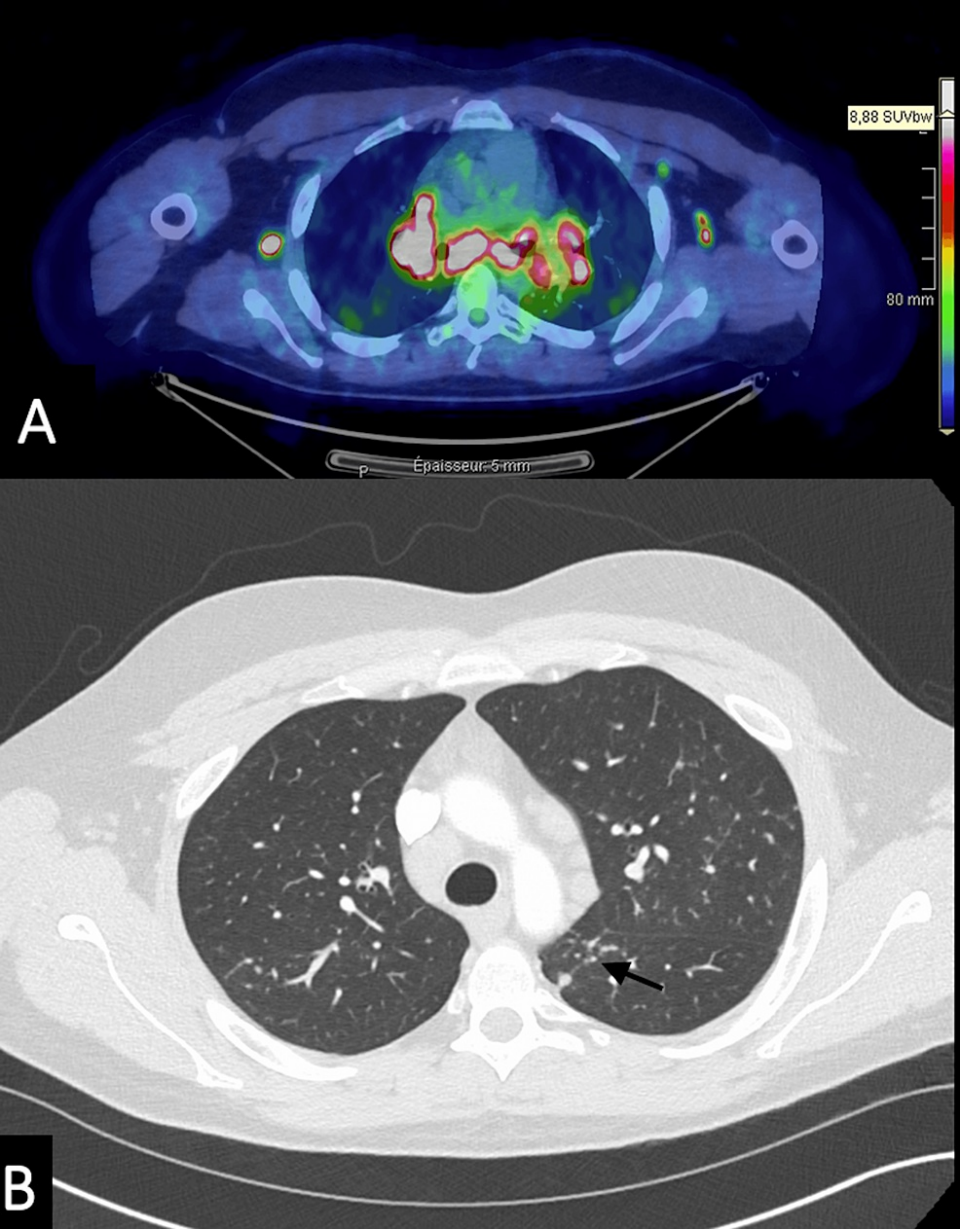
Multiple mediastinal, hilar, retroperitoneal, and inguinal hypermetabolic lymphadenopathies Axial FDG-PET/CT (A) shows in white mediastinal, hilar, and axillary lymphadenopathies with an elevated SUV. Axial chest CT (B) shows nodular and micronodular involvement of the subpleural and peribronchovascular regions in the upper segment of the left lower lobe (black arrow). The SUVmax was 8.88. FDG-PET/CT: fluorodeoxyglucose-positron emission tomography/computed tomography, SUV: standardized uptake value, CT: computed tomography, SUVmax: maximum standardized uptake value

In order to investigate these mediastinal lymphadenopathies, a thoracoabdominal CT scan was performed, as well as an FDG-PET/CT scan, to guide a potential biopsy. It showed multiple mediastinal, hilar, retroperitoneal, and inguinal hypermetabolic lymphadenopathies (Figure 3A), as well as typical pulmonary lesions of sarcoidosis (Figure 3B).

**Figure 4:**
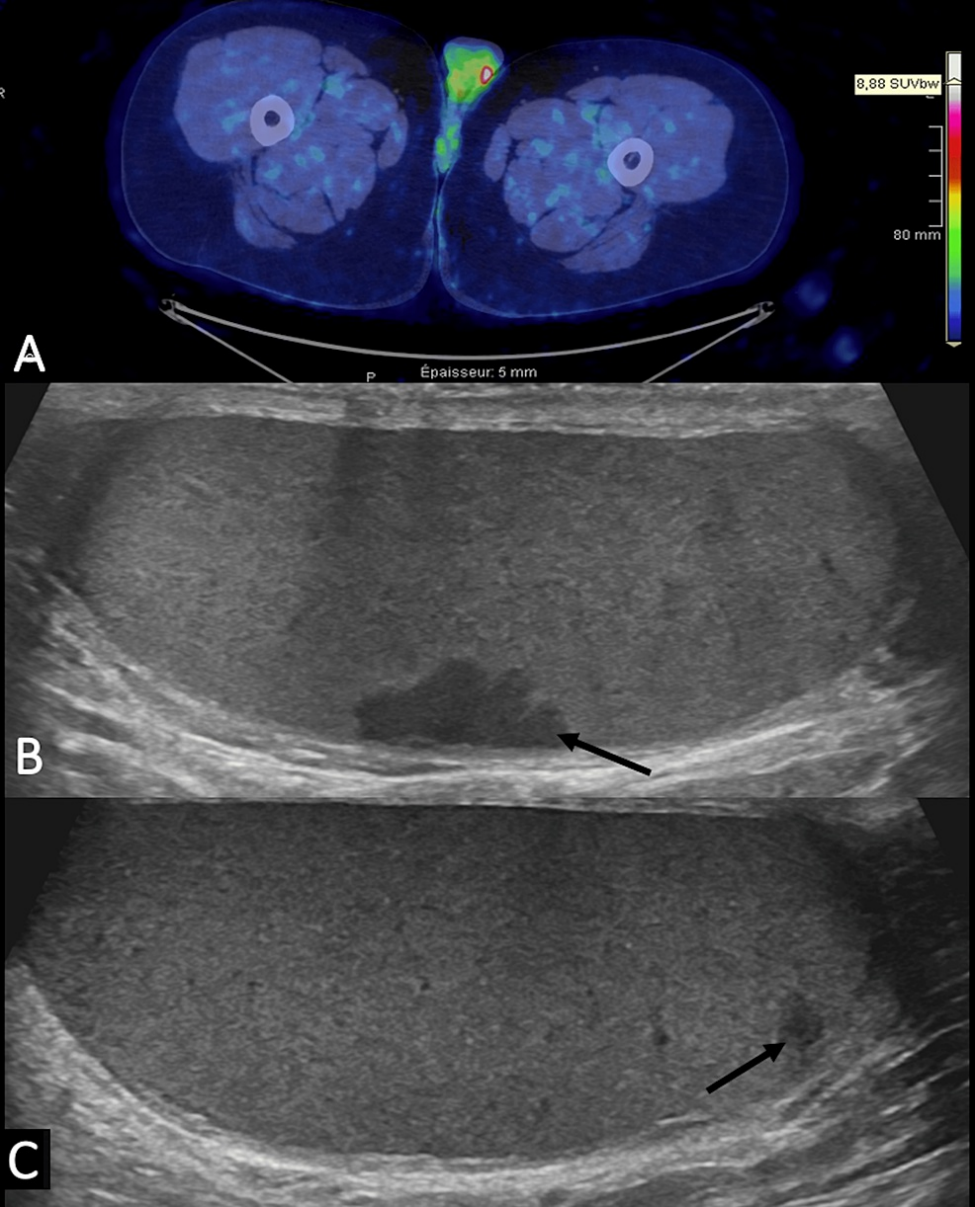
Axial FDG-PET/CT and scrotal ultrasonography Axial FDG-PET/CT (A) shows in white left testicular lesion with elevated SUV. Ultrasonography shows two nodular hypoechoic lesions (black arrows) in both testes appearing hypermetabolic on PET/CT, seen on the left (B) and the right (C). The SUVmax was 8.88. FDG-PET/CT: fluorodeoxyglucose-positron emission tomography/computed tomography, SUV: standardized uptake value, SUVmax: maximum standardized uptake value

FDG-PET/CT also showed hypermetabolic testicular lesions (Figure 4A). Testicular ultrasound showed bilateral hypoechoic poorly vascularized lesions (Figure 4B, 4C).

The patient underwent an ultrasound-guided lymph node biopsy, which showed non-caseating granulomas. This finding in the setting of negative tuberculosis and fungal studies was consistent with the diagnosis of sarcoidosis.

**Figure 5:**
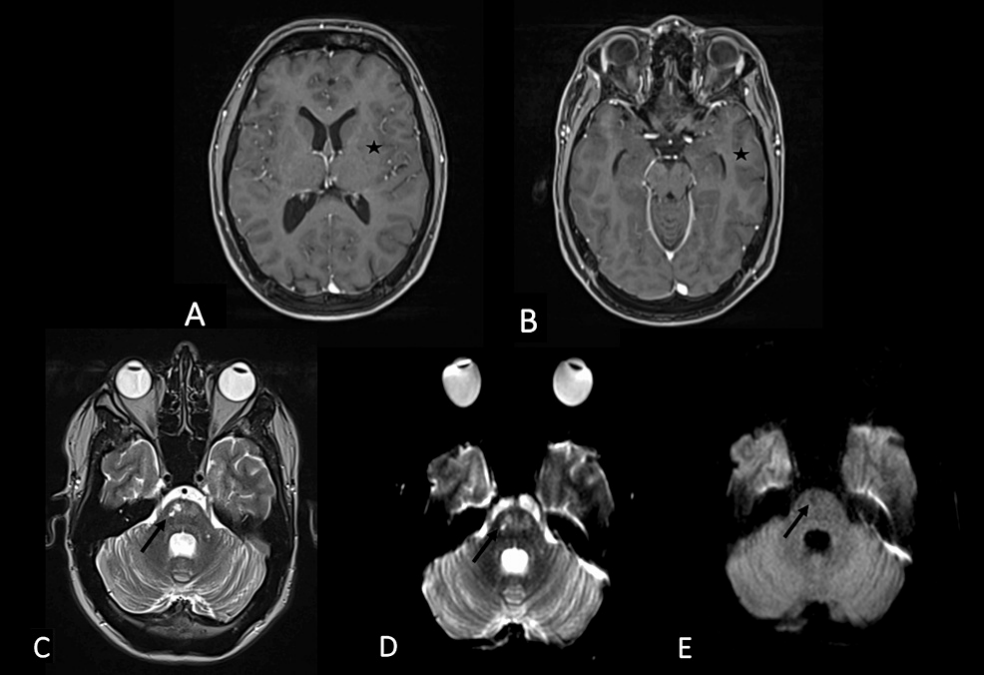
Brain MRI after three months of treatment MRI showing complete resolution of the leptomeningeal nodular enhancement after treatment (black stars) displayed on post-contrast axial T1WI (A,B). Pontic and brain stem chronic ischemic lesions are demonstrated (black arrows), characterized by a hypersignal on sequences axial T2 (C) without diffusion restriction (D,E). MRI: magnetic resonance imaging, T1WI: T1-weighted image

Corticosteroid and methotrexate treatments were initiated. After three months of therapy, a brain MRI demonstrated the resolution of the leptomeningeal nodular enhancement (Figure 5A, 5B) and showed progression to the chronicity of the previously reported pontine ischemic lesions (Figure 5C-5E). With ongoing treatment, significant neurological improvement was noted, including the resolution of headaches and dysarthria. However, gait ataxia persisted, although it showed signs of improvement.

**Figure 6:**
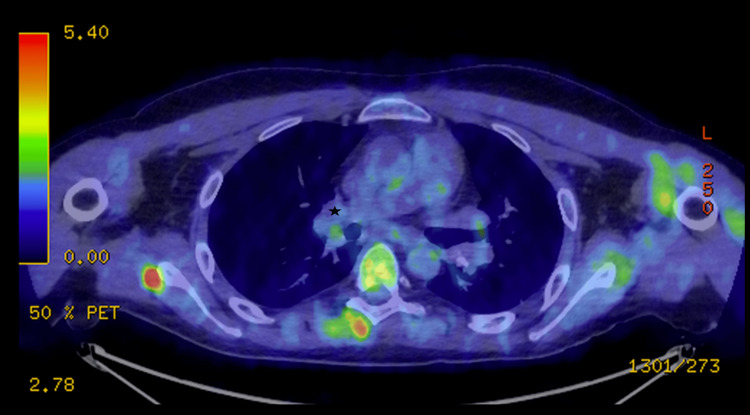
FDG-PET/CT after three months of treatment Axial FDG-PET/CT showed complete remission of mediastinal, hilar, and hypermetabolic lymphadenopathies (black star). FDG-PET/CT: fluorodeoxyglucose-positron emission tomography/computed tomography

A three-month follow-up FDG-PET/CT scan revealed a reduction in the hypermetabolism of the initially observed lymphadenopathies, which confirms a response to treatment (Figure 6).

## Discussion

Neurosarcoidosis has a diverse clinical spectrum, ranging from cranial neuropathies to myelopathies. Our patient's presentation with a combination of headaches, cerebellar dysarthria, and gait ataxia, against the backdrop of spontaneous remission from a similar episode a year prior, heightened the complexity of the diagnosis.

Headaches can be a potential symptom of neurosarcoidosis and may warrant further evaluation especially if other symptoms or physical findings are present. The diagnosis and evaluation of headaches typically involve a combination of medical history, clinical evaluation, and imaging studies [5,6]. In the case discussed, the headaches progressively worsened and intensified over 48 hours. Concurrently, stroke-like symptoms, such as dysarthria and gait disorders, further underscored the indication of brain imaging. The result was the discovery of leptomeningeal nodular enhancement (LNE).

The presence of LNE in neurosarcoidosis is generally associated with a more severe and progressive disease course and may be a poor prognostic indicator, especially when LNE affected blood flow to the brain or spinal cord and potentially lead to ischemia [7]. While neurosarcoidosis can involve various regions of the nervous system, involvement of the pons is considered an unusual presentation [8]. Gait disorders in neurosarcoidosis can be caused by damage to the parts of the brain or nervous system responsible for balance and coordination, such as cerebellum involvement, causing ataxia and cerebellar dysmetria [7]. Other symptoms of cerebellar dysfunction that occur in neurosarcoidosis include nystagmus and dysarthria [9]. Pontine ischemia can cause a variety of neurological symptoms, including anisocoria, gait disorders, and cerebellar dysarthria [10]. The precise mechanism by which pontine ischemia can lead to anisocoria is not well understood, but it may be related to damage to the neural pathways that control the size of the pupils [10].

There is considerable variation in the reported prevalence of LNE among neurosarcoidosis patients. Some studies suggest a frequency of 40%-50%, while others report a range from 10% to 40% [11-14]. The causes of LNE are varied, and in the case reported, differential diagnosis was metastatic cancer, lymphoma, meningitidis, and other inflammatory disorders of the nervous system, such as primary angiitis, ANCA vasculitis, and IgG4-related disease [12,13]. To further refine the differential diagnosis, blood tests and a lumbar puncture with CSF analysis and culture were performed and showed no signs of infection or neoplastic cells or findings suggestive of ANCA or IgG4 inflammatory disease. In our case, CSF analysis revealed lymphocyte-predominant pleocytosis with no elevated ACE. This marker is not necessarily raised in neurosarcoidosis, as in the case of our patient.

FDG-PET/CT played a dual role in this case. It not only unveiled systemic involvement, especially the characteristic pulmonary manifestations of sarcoidosis, but also guided biopsy decisions. The maximum standardized uptake values (SUVmax) provide a quantitative measure that can be helpful in monitoring disease activity and response to therapy [15]. One of the fundamental assumptions that guided the diagnostic process for sarcoidosis was the exclusion of other causes of granulomatous inflammation and the absence of *Mycobacterium tuberculosis*, fungi diseases, and neoplastic cells. We can confirm the diagnosis after the histopathologists showed the presence of non-caseating granulomas on biopsy in these conditions.

In some studies, the incidence of reproductive tract involvement in sarcoidosis has been reported to be less than 1% [15,16]. In males, sarcoidosis can affect the testes, epididymis, prostate gland, and seminal vesicles, with symptoms such as testicular pain, swelling, and infertility [15-17]. Enlarged lymph nodes in the groin area may also be present [18]. The exact mechanism of granuloma formation in the scrotum is not clear, but it is thought to involve a similar inflammatory response as in other affected tissues [16]. While scrotal involvement can occur in sarcoidosis, it is less common and typically manifests as skin lesions rather than nodular masses within the scrotal tissues. Ultrasonography is a valuable diagnostic tool that may show thickening or nodularity of the skin in the scrotal area, enlargement of the lymph nodes, and nodular lesions in the scrotal tissues, but the underlying tissues may appear relatively normal [16-18].

The treatment of multisystemic sarcoidosis involves a combination of medication and supportive therapies. The goal of treatment is to reduce inflammation and control symptoms, as well as to prevent complications and long-term damage to affected organs [17]. The specific treatment regimen will depend on the individual patient's needs and the severity and extent of the disease, as well as other individual factors such as age, overall health, and other medical conditions [5]. In many cases, corticosteroids, such as prednisone, are the first line of treatment and work by reducing inflammation and suppressing the immune system. Depending on the severity of the disease, and typically for neurosarcoidosis, higher doses may be necessary, and treatment may need to be continued for several months or longer [1-5]. Other immunosuppressive medications, such as methotrexate, azathioprine, or mycophenolate mofetil, may also be used [3]. In more severe cases of neurosarcoidosis, such as those involving compression of the spinal cord or brain stem, surgical intervention may be necessary.

The positive outcome observed in our patient, following the combined treatment of corticosteroids and methotrexate, aligns with current recommendations for managing neurosarcoidosis, in the case of severe involvement of CNS. The three-month follow-up MRI indicated effective disease control, showing a reduction in leptomeningeal nodular enhancement. Furthermore, the three-month follow-up FDG-PET/CT revealed a favorable evolution of the previously detected hypermetabolic abnormalities, such as the lymphadenopathies. However, persistent gait ataxia is a reminder of the possibility of long-term impairment, underlining the importance of rapid diagnosis and treatment.

## Conclusions

In summary, this case illustrates an unusual form of neurosarcoidosis with multi-organ involvement, which raised the question of differential diagnosis of neoplastic disease and tuberculosis. The diagnosis was made with a lymph node biopsy that showed a non-caseating granuloma without necrosis and the exclusion of mycobacterium tuberculosis, fungi, and other causes. Overall, this case report highlights the importance of a multidisciplinary approach to the diagnosis and management of sarcoidosis.
